# Efficacy of Acupoint Application on *In Vitro* Fertilization Outcome in Patients with Polycystic Ovary Syndrome: A UHPLC-MS-Based Metabolomic Study

**DOI:** 10.1155/2022/9568417

**Published:** 2022-10-14

**Authors:** Lingyu Yu, Qingchang Xia, Zhengao Sun, Jingyan Song

**Affiliations:** ^1^Institute of Chinese Medical Literature and Culture, Shandong University of Traditional Chinese Medicine, Jinan, China; ^2^College of Acupuncture and Massage, Shandong University of Traditional Chinese Medicine, Jinan, China; ^3^Reproductive and Genetic Center of Integtated Traditional and Western Medicine, Affiliated Hospital of Shandong University of Traditional Chinese Medicine, Jinan, China

## Abstract

**Objective:**

To explore the clinical effect of acupoint application on the outcome of *in vitro *fertilization-embryo transfer (IVF-ET) in patients with polycystic ovary syndrome (PCOS) of the phlegm-dampness type and elucidate its possible mechanism of action from the perspective of follicular fluid metabolomics.

**Methods:**

A total of 90 patients undergoing IVF-ET due to infertility were selected and divided into three groups: the treatment group (PCOS with acupoint application, *n* = 30), the control group (PCOS without acupoint application, *n* = 30), and the normal group (non-PCOS, *n* = 30). All patients received a gonadotropin-releasing hormone agonist (GnRH-a) long protocol for controlled ovarian hyperstimulation (COH). Among them, the treatment group was also given the acupoint application from the day of pituitary downregulation to the day of the human chorionic gonadotrophin (hCG) trigger. Ultrahigh-performance liquid chromatography connected with quadrupole time-of-flight mass spectrometry (UHPLC-MS) was adopted for untargeted metabolomic analysis of follicular fluid collected from the three groups of patients on the day of oocyte pick-up (OPU). The significantly differential metabolites were screened using univariate and multivariate statistical analysis, and the related metabolic pathways were identified by the Kyoto Encyclopedia of Genes and Genomes (KEGG) pathway analysis.

**Results:**

Metabolomic analysis showed that the treatment group's follicular fluid samples were aggregated with the normal group and separated from the control group. A total of 34 significantly differential metabolites were found in the follicular fluid of patients with phlegm-dampness PCOS and normal people. With the intervention of acupoint application, seven metabolites (pseudouridine, phenol, 2-oxoadipic acid, 9R,10S-EpOME, DL-lactate, nicotinamide, and DL-indole-3-lactic acid) were all downregulated, mainly involving the pathways of pyruvate metabolism, nicotinate and nicotinamide metabolism, protein digestion and absorption, biosynthesis of amino acids, and pyrimidine metabolism.

**Conclusions:**

Acupoint application can effectively improve the clinical symptoms and the outcome of IVF-ET treatment in patients with PCOS of the phlegm-dampness type, and its mechanism of action may be related to the regulation of the pathways of pyruvate metabolism, nicotinate and nicotinamide metabolism, protein digestion and absorption, biosynthesis of amino acids, and pyrimidine metabolism.

## 1. Introduction

Polycystic ovary syndrome (PCOS) is a complex endocrine disease caused mainly by genetic and environmental factors and is often accompanied by reproductive dysfunction and abnormal glucose and lipid metabolism. It is characterized by oligoovulation or nonovulation, androgen excess, and polycystic changes in the ovaries. Notably, 70∼80% of PCOS patients are often associated with infertility [1]. For PCOS patients who wish to conceive, the common clinical treatment drugs are letrozole, clomiphene, or gonadotropin (Gn). In addition, *in vitro *fertilization-embryo transfer (IVF-ET) technology needs to be applied for infertile PCOS patients due to ovulation induction failure, pure spouse problems, or fallopian tube factor. However, although IVF-ET technology can make PCOS patients produce more oocytes, embryo quality and pregnancy outcomes are often not optimal. Therefore, how to solve the above problems has become the focus of the current research.

A previous study revealed that about 30∼70% of PCOS patients were overweight or obese [2]. Traditional Chinese medicine believes that phlegm dampness is the most common type of PCOS [3] and provides some effective treatment methods, including traditional Chinese medicine decoction, acupuncture, moxibustion, acupoint application, acupoint catgut embedding, and others [4]. Among them, the acupoint application is a widely used external therapy and is based on the holistic concept and dialectical treatment of traditional Chinese medicine. In the first step, Chinese herbal medicines are processed into powder and mixed with excipients such as water or honey to create ointments, followed by applying them to specific acupoints on the body surface. Acupoint application can treat diseases from *in vitro* to *in vivo* by transdermal absorption of traditional Chinese medicine ingredients and stimulating acupoints on the body surface. Therefore, it has the dual functions of Chinese herbal medicines and acupoints, and its selection of Chinese herbal medicines and acupoints also varies with diseases and syndromes. Compared to other traditional Chinese medicine therapies, acupoint application has the advantages of zero suffering, low cost, simple operation, high safety factor, and not requiring special medical equipment or instruments.

Metabolomics, first proposed by Professor Nicholson in 1999 [5], can analyze biomarkers from a macroperspective, thereby reflecting the internal state of organisms more directly and accurately. Metabolomics is divided into two categories: untargeted analysis and targeted analysis. The untargeted metabolomics is usually established on a high-resolution mass spectrometer, relying on its powerful high-resolution mass analyzer, which can conduct nonbiased, large-scale, and systematic detection of various metabolites in the sample, thus comprehensively and systematically reflecting the metabolic characteristics of the living bodies. Therefore, it is suitable for basic research in the early stage of a project. Sun et al. [6] analyzed the follicular metabolomic changes in the ovaries of mice based on the ultrahigh-performance liquid chromatography/mass spectrometry (UPLC-MS) technique, which provided new insight into understanding follicular development. Recently, metabolomics has already shown potential in identifying underlying biomarkers in both human and animal models of PCOS and has played a role in studying and evaluating the mechanisms of PCOS [7].

Follicular fluid is composed of plasma exudate and ovarian partial secretions that contain a variety of metabolites associated with the development and maturation of follicles. Our previous research found that women with PCOS displayed unique metabolic profiles in their follicular fluid by using an advanced sequential window acquisition of all theoretical fragment-ion spectra (SWATH) mass spectrometry, the components of which might be potentially applied as biomedical markers for diagnosing PCOS [8]. In addition, analyzing metabolites in the follicular fluid of PCOS patients can also help to evaluate the growth and development potential of oocytes, which will aid in predicting pregnancy outcomes [9].

This study took IVF-ET technology as the platform, follicular fluid as the research object, and acupoint application therapy as the intervention method and focused on infertile patients with PCOS of phlegm-dampness type. Using the UHPLC-MS-based metabolomic analysis technique, we observed changes in follicular fluid metabolites in patients with PCOS of a phlegm-dampness type after the acupoint application. The study aimed to identify the characteristic metabolites of PCOS of the phlegm-dampness type, reveal the mechanism of acupoint application, and explore more effective treatment methods for improving IVF-ET outcomes of PCOS infertility patients.

## 2. Materials and Methods

### 2.1. Materials

#### 2.1.1. Preparation of Acupoint Application

*Atractylodis Rhizoma*, *Cyperi Rhizoma*, *Aurantii Fructus*, *Citrus Reticulata Pericarpium*, *Poria*, *Rhei Radix Et Rhizoma*, *Magnoliae Officinalis Cortex*, *Pinelliae Rhizoma*, *Arisaematis Rhizoma Praeparatum*, *Glycyrrhizae Radix et Rhizoma*, *Trichosanthes Kirilowii Maxim Fructus*, and *Sinapis Semen* were the Chinese medicinal herbs selected for the preparation of ointments. Furthermore, these herbs were crushed into powder using a pulverizer, proportionally mixed at a ratio of 6 : 6: 6 : 4: 4 : 4: 4 : 3: 3 : 3: 3 : 2, and blended with refined honey to create ointments. The ointments were manufactured in the Chinese Pharmacy Department of the Affiliated Hospital of Shandong University of Traditional Chinese Medicine, authenticated by Dr. Sun (Affiliated Hospital of Shandong University of Traditional Chinese Medicine, Jinan, China), and the regulatory guidance requirements issued by the China Food and Drug Administration were met. Approximately 3 g of ointment was squeezed onto a circular external nonwoven breathable adhesive fabric (Haishi Hainuo Beishiwei Medical Products Co., Ltd.; product specification: 6 × 6 cm, inner diameter: 2 cm). These nonwoven fabrics with ointments would be stuck to acupoints on the body surface, including Fenglong point (ST40), Yinlingquan point (SP9), Zusanli point (ST36), Zhongwan point (CV12), Shenque point (CV8), Guanyuan point (CV4), Tianshu point (ST25), and Daimai point (GB26).

#### 2.1.2. Experimental Equipment

*(1) Experimental Instruments and Reagents*. The following instruments and reagents were used: AB Triple TOF 6600 mass spectrometer (AB SCIEX); Agilent 1290 Infinity LC ultrahigh-pressure liquid chromatograph (Agilent); low-temperaturehigh-speed centrifuge (Eppendorf 5430 R); chromatographic column: Waters, ACQUITY UPLC BEH Amide 1.7 *μ*m, 2.1 mm × 100 mm column; acetonitrile (Merck, 1499230–935); ammonium acetate (Sigma,70221); and ammonia water (Sigma, 221228–500 ML).

*(2) Chromatography-Mass Spectrometry Analysis*.

   Chromatography conditions.
  Samples were separated using the Agilent 1290 Infinity LC UHPLC with HILIC column. Column temperature: 25°C; flow rate: 0.5 mL/min; and sample injection volume: 2 *μ*L. Mobile phase A: water with 25 mM ammonium acetate and 25 mM ammonia water; mobile phase B: acetonitrile. The gradient elution procedure was as follows: 0∼0.5 min, 95% B; 0.5∼7 min, B changed linearly from 95% to 65%; 7∼8 min, B changed linearly from 65% to 40%; 8∼9 min, B maintained at 40%; 9∼9.1 min, B changed linearly from 40% to 95%; and 9.1∼12 min, B maintained at 95%. Notably, the samples were placed in an autosampler at 4°C throughout the analysis. To avoid adverse consequences caused by fluctuations of instrument signals, a random sequence was adopted to maintain continuous analysis of samples. The system's stability and the experimental data's reliability could be judged by inserting the quality control (QC) samples into the sample queue.
  Q-TOF mass spectrometry conditions.
  The AB Triple TOF 6600 mass spectrometer was used to collect the primary and secondary spectra of the samples. The electrospray ionization (ESI) source conditions after HILIC chromatographic separation were as follows: ion source Gas2 (Gas2): 60, curtain gas (CUR): 30, source temperature: 600°C, ion spray voltage floating (ISVF) ± 5500V (both positive and negative modes); TOF MS scan *m/z* range: 60∼1000 Da; product ion scan *m/z* range: 25∼1000 Da; TOF MS scan accumulation time: 0.20 s/spectra; and product ion scan accumulation time: 0.05 s/spectra. Secondary mass spectrometry was obtained using information-dependent acquisition (IDA) and high sensitivity mode. Declustering potential (DP): ±60 V (both positive and negative modes); collision energy: 35 ± 15 eV. IDA was set as follows: exclude within 4 Da; candidate ions to monitor per cycle: 10.

### 2.2. Subjects

This study was approved by the Reproductive Medicine Ethics Committee of the Affiliated Hospital of Shandong University of Traditional Chinese Medicine and registered with the China Clinical Trials Registration Center (Registration No.: ChiCTR1900026889). All subjects signed a written informed consent form prior to the study.

A total of 60 patients with phlegm-dampness PCOS undergoing IVF-ET at the Reproductive and Genetic Center of Integrated Traditional and Western Medicine, Affiliated Hospital of Shandong University of Traditional Chinese Medicine from November 2019 to September 2020 were included and distributed into two groups, namely, the treatment group (30 cases) and the control group (30 cases). Another 30 healthy women who underwent IVF-ET purely due to spouse problems were selected as the normal group. The treatment group was treated with acupoint application on the basis of routine controlled ovarian hyperstimulation (COH) from the day of pituitary down-regulation to the day of human chorionic gonadotrophin (hCG) trigger, once a day for 6 h.

The inclusion criteria for the treatment group and the control group are as follows: (1) meeting the diagnostic criteria of PCOS and infertility; (2) meeting the traditional Chinese medicine dialectical standard of phlegm-dampness type; (3) age between 21 and 35 years; (4) having basically normal semen analysis of spouse; and (5) adopting gonadotropin-releasing hormone agonist (GnRH-a) long protocol for COH. The exclusion criteria included the following: (1) suffering from infectious diseases, hepatorenal insufficiency, cardiovascular diseases, mental diseases, and other endocrine diseases besides PCOS; (2) suffering from endometriosis, uterine fibroids, and other gynecological diseases; (3) previous history of ovarian surgery, radiotherapy, or chemotherapy; (4) skin ulceration; and (5) severe allergic constitution or recent history of infection. Notably, the normal group included healthy women who underwent IVF or intracytoplasmic sperm injection (ICSI) purely due to spouse problems at the age of 21∼35 years.

All patients were treated with GnRH-a long protocol. Triptorelin acetate (a kind of GnRH-a; Ferring Pharmaceuticals, Saint-Prex, Switzerland) was subcutaneously injected with 0.05 mg per day from the middle of the luteal phase of the last menstrual cycle. When reached the standard of pituitary down-regulation, exogenous Gn: follicle-stimulating hormone (FSH; Merck Serono SA Aubonne Branch) and human menopausal gonadotropin (hMG; Zhuhai Lizhu group Libao Biochemical Pharmaceutical Co., Ltd) were administered. The growth of follicles was monitored by transvaginal ultrasound and sex hormone level, and the dosage of Gn was properly adjusted according to the number and growth rate of follicles. 10,000 U of human chorionic gonadotropin (hCG; Zhuhai Lizhu group Libao Biochemical Pharmaceutical Co., Ltd) was injected intramuscularly when two follicles with diameter ≥18 mm or three follicles with diameter ≥17 mm, and the estradiol (*E*_2_) level of each dominant follicle (diameter ≥14 mm) reached 200∼300 pg/mL. Next, oocytes were collected 35∼36 h after hCG injection using transvaginal ultrasound-guided ovarian puncture. After collection, oocytes were cultured in a 5% CO_2_ incubator before fertilization, and the appropriate method to deal with the semen was selected according to the specific conditions of the semen on the same day. The oocytes were fertilized according to the motile sperm concentration of 10^5^ sperm/ml, followed by either the transfer of the embryos into the uterine cavity or freezing on day three after oocyte pick-up (OPU).

The first tube of yellowish, bloodless, and transparent follicular fluid was collected on the day of OPU, and the presence of mature oocytes was confirmed under a microscope. After centrifugation (3000 g, 15 min), the supernatant was labeled and stored at −80°C until further analysis. Next, UHPLC-Q-TOF MS was used to detect the metabolites in the samples, and the metabolites in the biological samples were identified by matching the metabolite information in the local database.

### 2.3. Sample Extraction Method

After the sample was slowly thawed at 4°C, the appropriate amount of sample was added to the precooled methanol/acetonitrile/aqueous solution (2 : 2 : 1, v/v) and vortexed to mix. Next, the sample was subjected to low-temperature ultrasound for 30 min, allowed to stand at −20°C for 10 min, and centrifuged at 14000 g for 20 min at 4°C. The supernatant was then dried in a vacuum, and 100 *μ*L acetonitrile aqueous solution (acetonitrile: water = 1 : 1, v/v) was added to redissolve while mass spectrometry analysis was performed and vortexed to mix. Finally, the sample was centrifuged at 14000 g for 15 min at 4°C, and the supernatant was taken for sample analysis.

### 2.4. Experimental Data Analysis Process

The original data in Wiff format were converted into .mzXML format by ProteoWizard. Next, peak alignment, retention time correction, and peak area extraction were performed using the XCMS software. The data extracted by XCMS were first subjected to metabolite structure identification and data preprocessing, and then, the quality experimental data were evaluated. Finally, we performed data analysis, including univariate statistical analysis, multivariate statistical analysis, screening for differential metabolites, and Kyoto Encyclopedia of Genes and Genomes (KEGG) pathway analysis.

### 2.5. Quality Evaluation of Experimental Data

The experimental data were evaluated using the relative standard deviation (RSD) of QC samples. It is worth noting that the smaller the RSD of the ion peak abundance of QC samples, the better the stability of the instrument. The peak number of RSD ≤30% accounted for more than 80% of the total peak number of QC samples, indicating that the stability of the instrument analysis system was good and the data could be used for subsequent analysis.

### 2.6. Statistical Analysis

All statistical analyses were performed using IBM SPSS statistics 26.0 software, and all measurement data were expressed as mean ± standard deviation. One-way analysis of variance (ANOVA) was used for comparison if the three groups of data conformed to the normal distribution; otherwise, the rank sum test was used. In instances where the total difference between groups was statistically significant, pairwise comparison was used. Moreover, the paired *t*-test was used for intragroup comparison and count data were statistically described by constituent ratios using the chi-square test. *P* < 0.05 was considered statistically significant.

## 3. Results

### 3.1. Clinical Background

There was no significant difference in age, years of infertility, basic FSH (bFSH), basic *E*_2_ (bE_2_), basic progesterone (bP), and basic testosterone (bT) among the three groups (*P* > 0.05). There was no significant difference in body mass index (BMI), menstrual cycle, basic luteinizing hormone (bLH), and basic LH/FSH (bLH/FSH) between the treatment group and the control group (*P* > 0.05), but all were significantly higher than those in the normal group (*P* < 0.05), as shown in Table 1.

### 3.2. COH Results

The results showed that there was no significant difference in total Gn, *E*_2_, P, and LH levels on hCG day, number of high-quality embryos, embryo implantation rate, and cumulative pregnancy rate among the three groups (*P* > 0.05). Gn days in the treatment group were less than those in the control group (*P* < 0.05). The number of retrieved oocytes and fertilized oocytes in the treatment group was higher than that in the normal group (*P* < 0.05), and the number of available embryos in the treatment group was higher than that in the control group and the normal group (*P* < 0.001). It is suggested that acupoint application can improve the quality of oocytes in patients with PCOS, effectively promote follicular growth, and then improve the outcome of IVF to some extent, as shown in Table 2.

### 3.3. Traditional Chinese Medicine Syndrome Integral

After the intervention of acupoint application, the phlegm-dampness syndrome integral of the treatment group on hCG day was significantly lower than that on the downregulation day (*P* < 0.001). It is suggested that acupoint application can improve the phlegm-dampness syndrome of PCOS patients, as shown in Table 3.

### 3.4. Statistics and Analysis of Metabolic Identification Results

#### 3.4.1. Metabolite Identification Grade

This study used the local self-built standard database of Zhongke New Life (in-house database (Shanghai Applied Protein Technology)) to search the database. By matching with the retention time, molecular mass (molecular mass error < 25 ppm), secondary order fragmentation spectrum score > 0.7, and collision energy of the metabolites in the local database, the structure of the metabolites in biological samples was identified and the identification results were strictly checked and confirmed manually. The identification grade is above Level 2.

#### 3.4.2. Statistics of Chemical Classification of Metabolites

**Figure 1:**
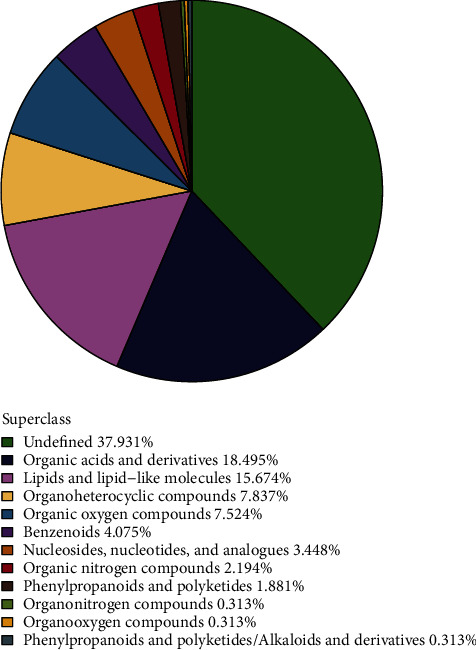
Proportion of identified metabolites in chemical categories. Note: the color blocks of different colors in the figure expressed different chemical classification attribution entries, and the percentage represented the percentage of the number of metabolites in the attributive entry of the chemical classification to the total number of metabolites identified. The metabolites without chemical classification were presented as undefined.

A total of 319 metabolites were identified after the combination of positive and negative ion modes. These metabolites were classified and counted, mainly including organic acids and derivatives, lipids and lipid-like molecules, organoheterocyclic compounds, organic oxygen compounds, benzenoids, nucleosides, nucleotides and analogues, and organic nitrogen compounds. The proportions of various metabolites are shown in Figure 1.

### 3.5. Between-Group Difference Analysis

#### 3.5.1. Univariate Statistical Analysis

**Figure 2:**
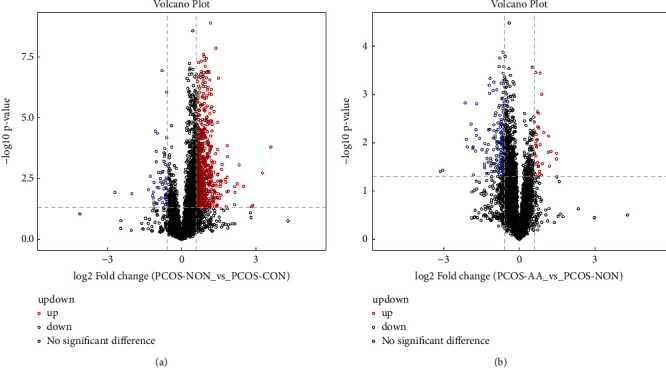
Volcano map in negative ion mode. (a) Intervention without acupoint application. (b) Intervention with acupoint application. Note: the abscissa is the logarithm of log2 of FC, and the ordinate is the logarithm of -log10 of significant *P* value. PCOS-AA: treatment group; PCOS-NON: control group; PCOS-CON: normal group.

Differential analysis was performed for all metabolites (including unidentified metabolites) detected in positive and negative ion modes using univariate analysis. Differential metabolites with fold change (FC) > 1.5 or FC < 0.67 and *P* value < 0.05 were visualized in the form of volcano maps. Taking the negative ion mode as an example, it is suggested that acupoint application can cause the change of upregulation and downregulation of the follicular fluid metabolites of PCOS patients from Figure 2.  Figure 2(a) shows that the metabolites are mainly upregulated without the intervention of acupoint application, and Figure 2(b) shows that the metabolites are mainly downregulated with the intervention of acupoint application, which indicates that acupoint application can change the follicular fluid metabolites of PCOS patients.

#### 3.5.2. Multivariate Statistical Analysis

**Figure 3:**
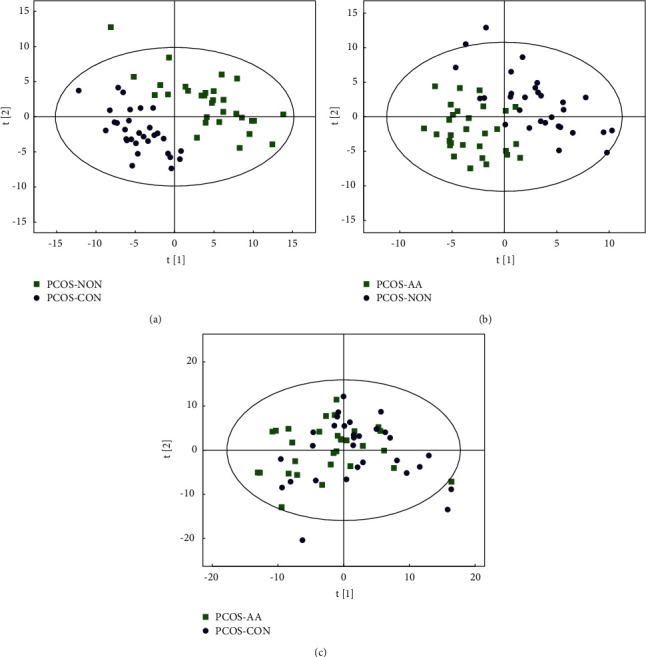
PLS-DA model scores. Note: PCOS-AA: treatment group; PCOS-NON: control group; PCOS-CON: normal group.

The partial least squares discriminant analysis (PLS-DA), a supervised discriminant analysis statistical method which uses partial least squares regression, was applied to establish a relationship model between metabolite expression quantity and sample category with the goal of predicting sample category. Through the established discriminant model, differential lipids associated with grouping could be screened from the data set.  Figure 3 shows that the PLS-DA model can distinguish each group of samples: Figure 3(a) shows the separation of samples between the control group and the normal group; Figure 3(b) shows the separation of samples between the treatment group and the control group; Figure 3(c) shows the aggregation of samples between the treatment group and the normal group. It is suggested that there is a significant difference in follicular fluid metabolites between PCOS patients and normal people and acupoint application can reduce the difference and make the follicular fluid metabolites of PCOS patients closer to normal people.

#### 3.5.3. Screening Significantly Differential Metabolites

Orthogonal partial least squares discriminant analysis (OPLS-DA) VIP >1 and *P* value < 0.05 were used as the cut-off values for screening significantly differential metabolites. A total of 72 significantly differential metabolites were identified: 25 between the treatment group and the normal group, of which 19 were upregulated and 6 were downregulated; 34 between the control group and the normal group, of which 33 were upregulated and 1 was downregulated; and 13 between the treatment group and the control group, of which 4 were upregulated and 9 were downregulated, as shown in Table 4.

### 3.6. Analysis of Differential Metabolites

#### 3.6.1. Differential Metabolites between PCOS of Phlegm-Dampness Type and Normal People

**Figure 4:**
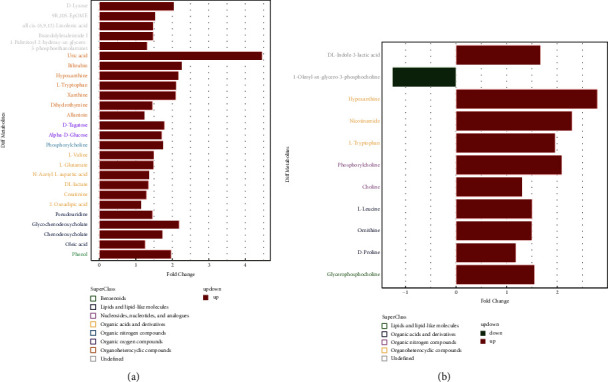
Differential metabolites between PCOS of phlegm-dampness type and normal people. (a) Negative ion mode; (b) positive ion mode.

Figure 4 shows details of the significantly differential metabolites between patients with PCOS of the phlegm-dampness type and normal people in positive and negative ion modes. Among them, the upregulated metabolites included 2-oxoadipic acid, *D*-lyxose, pseudouridine, phosphorylcholine, *L*-valine, *D*-tagatose, *alpha-D*-glucose, *L*-tryptophan, xanthine, *DL*-lactate, *L*-glutamate, dihydrothymine, creatinine, phenol, *all cis*-(6,9,12)-linolenic acid, bisindolylmaleimide I, bilirubin, hypoxanthine, 9*R*,10*S*-EpOME, N-acetyl-L-aspartic acid, uric acid, oleic acid, allantoin, chenodeoxycholate, glycochenodeoxycholate, 1-palmitoyl-2-hydroxy-sn-glycero-3-phosphoethanolamine, choline, glycerophosphocholine, *DL*-indole-3-lactic acid, *L*-leucine, nicotinamide, ornithine, and *D*-proline. On the other hand, the downregulated metabolite was 1-oleoyl-sn-glycero-3-phosphocholine.

#### 3.6.2. Changes in Significantly Differential Metabolites

**Figure 5:**
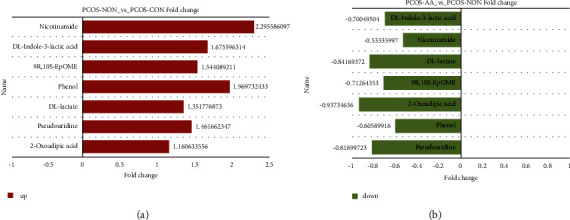
Changes in multiples of differential metabolite expression. (a) Intervention without acupoint application. (b) Intervention with acupoint application. Note: PCOS-AA: treatment group; PCOS-NON: control group; PCOS-CON: normal group.

With the intervention of acupoint application, seven metabolites (pseudouridine, phenol, 2-oxoadipic acid, 9*R*, 10*S*-EpOME, *DL*-lactate, nicotinamide, and *DL*-indole-3-lactic acid) changed significantly. Notably, the common feature was that they all changed from upregulation, as in Figure 5(a), to downregulation, as in Figure 5(b).

#### 3.6.3. Heatmap Analysis of Significantly Differential Metabolites

**Figure 6:**
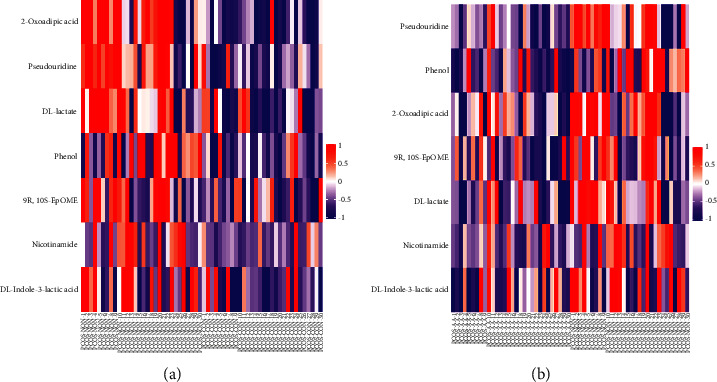
Heatmaps of seven significantly differential metabolites. (a) Control group vs. normal group; (b) treatment group vs. control group. Note: PCOS-AA: treatment group; PCOS-NON: control group; PCOS-CON: normal group. Each row represented a differential metabolite (the ordinate was the metabolite of significant differential expression), and each column represented a set of samples (the abscissa is the sample information). Red represented significant upregulation, blue represented significant downregulation, and color depth represented the degree of upregulation or downregulation.

Figure 6 shows the different distribution patterns of the seven significantly differential metabolites in each follicular fluid sample of the three groups: These metabolites are mainly upregulated in the control group and downregulated in the treatment group and normal group. It is suggested that acupoint application can change the seven significantly differential metabolites, which indirectly indicates that acupoint application is effective in the treatment of PCOS patients.

### 3.7. KEGG Pathway Analysis

Results showed that the pathways of pyruvate metabolism, nicotinate and nicotinamide metabolism, protein digestion and absorption, biosynthesis of amino acids, and pyrimidine metabolism were regulated with the intervention of acupoint application, as shown in Table 5.

## 4. Discussion

To date, the exact pathogenesis of reproductive dysfunction in patients with PCOS is still unclear. Modern studies suggest that PCOS may result from the interaction of multiple factors such as adiposity and endocrine dysfunction, which form a vicious circle of abnormal follicular development, oocyte maturation disorder, abnormal steroid hormone synthesis, and decreased endometrial receptivity, thereby leading to ovarian and endometrial dysfunction [10]. Studies have revealed that PCOS is associated with adiposity, insulin resistance (IR), and glucose and lipid metabolism disorders [11], and the modern biological basis of phlegm-dampness is associated with IR and glucose and lipid metabolism disorders [12], which suggests that PCOS is closely associated with phlegm-dampness. Acupoint application has been widely used in the treatment of PCOS, where it plays an important role in adjusting the menstrual cycle and improving acne [13]. The meta-analysis described by Liu [14] and Huang [15] provided a detailed and reliable basis for the efficacy and safety of acupoint application. In this study, only one patient had an allergic skin reaction in the course of treatment, characterized by slight redness of the local skin and mild itching, and then returned to normal after stopping the application for several hours, which suggested that acupoint application had a high safety factor and few adverse reactions.

In this study, the selection of Chinese herbal medicine and acupoints strictly followed the principle of syndrome differentiation and treatment of traditional Chinese medicine. The phlegm-dampness syndrome integral of the treatment group was significantly lower on the day of hCG trigger than that on the day of downregulation (*P* < 0.001), indicating that acupoint application could significantly eliminate phlegm dampness and had good effects on the clinical treatment of patients with PCOS of phlegm-dampness type. The number of oocytes retrieved in the treatment group was similar to that in the control group (*P* > 0.05), but the number of available embryos in the control group was lower than that in the treatment group (*P* < 0.05). Therefore, we speculated that acupoint application might improve the quality of oocytes to some extent, thereby improving the quality of embryos. Although there was no significant difference in the number of high-quality embryos and the pregnancy outcome of IVF among the three groups (*P* > 0.05), the treatment group showed an improvement trend compared to the control group. It is suggested that acupoint application might improve the oocyte quality of patients with PCOS of the phlegm-dampness type in some aspects, thereby improving the pregnancy outcome. But its improvement effect was not obvious, which might be attributed to the small sample size and short intervention time of acupoint application.

Follicular fluid provides an important external environment for oocyte growth and development, and the change of its components can directly affect the quality of oocyte [16]. With the rapid development of metabonomics, follicular fluid has become an objective index to evaluate the quality of oocytes, which plays an important role in predicting the outcome of IVF. Metabonomics looks for related biochemical pathways by analyzing the changes in metabolites in follicular fluid [17]. Targeted metabolomics focuses on multiple analysis of known metabolites with high sensitivity but limited coverage. On the contrary, untargeted metabolomics is mainly to detect the differences in metabolites among groups, which is an aimless large-scale detection method and has broad coverage. Therefore, it can comprehensively and systematically reflect the characteristics of the metabolites and has the potential to determine new biomarkers [18]. Based on this, we chose untargeted metabolomics technology.

The results of metabolomics analysis showed that 34 significantly differential metabolites were found in the follicular fluid of patients with PCOS of phlegm-dampness type and normal people, mainly involving the biochemical pathways of amino acids, carbohydrates, lipids, and purine metabolism. Previous studies have shown that the metabolic characteristics of PCOS women mainly involve the metabolic disorders of amino acids, carbohydrates, steroid hormones, nucleosides, lipids, and purines, which is consistent with our findings, and it may be associated with IR, oxidative stress, and ovulatory dysfunction [19, 20].

After combining clinical data with metabolomics results, it was found that the symptoms of the phlegm-dampness in the treatment group were significantly improved and the clinical observation indexes were better than those in the control group, and the follicular fluid composition analysis in the treatment group was aggregated with that in the normal group but separated from that in the control group, which indicated that acupoint application had a certain effect that could cause a series of changes in the metabolites in the follicular fluid. After comparing the significantly differential metabolites among the three groups, we found that seven metabolites (pseudouridine, phenol, 2-oxoadipic acid, 9*R*,10*S*-EpOME, *DL*-lactate, nicotinamide, and *DL*-indole-3-lactic acid) had significant changes. The common feature was that they all changed from up-regulation to down-regulation with the intervention of acupoint application. The KEGG database is often used for pathway research, and KEGG pathway analysis can improve our understanding of the biological functions of metabolites. The results of KEGG pathway analysis showed that the seven metabolites were mainly involved in the pathways of pyruvate metabolism, nicotinate and nicotinamide metabolism, protein digestion and absorption, biosynthesis of amino acids, and pyrimidine metabolism.

Pyruvic acid, mainly derived from glycolysis, is one of the most important metabolites in cells. The increase of pyruvate metabolism in the ovary is associated with the gender difference in gonadal formation and the beginning of ovarian meiosis [21]. It has been found that PCOS can cause the disorder of pyruvate metabolism [22]. Our study found that *DL*-lactate in the pyruvate metabolic pathway changed with the intervention of acupoint application. The concentration of lactate in patients with PCOS is high, so it is often used as one of the compounds to identify PCOS [19]. And the high lactate signal was also an important pirce of evidence of the harmful effects on growing follicles [23]. Therefore, we speculate that the down-regulation of the *DL*-lactate level in the pyruvate metabolism pathway with the intervention of acupoint application may improve the quality of follicles to some extent.

Nicotinate and nicotinamide, collectively known as nicotinic acid (also known as vitamin B_3_), are central regulators of physiological processes such as genetic stability and can regulate the epigenetic control mechanism of metabolism and aging [24]. It has been found that nicotinamide can inhibit androgen synthesis and improve IR in patients with PCOS to some extent, but only at the optimal dose [25], which indicates that the dose of nicotinamide is a key factor in whether it can play a positive role in the treatment of PCOS. However, we have not found detailed reports on this issue.

It was reported that protein digestion and absorption seemed to be involved in ovarian development, and closely associated with energy metabolism, hormone synthesis and ovarian function [26, 27]. Our study found that phenol in the pathway of protein digestion and absorption was regulated from up-regulation to down-regulation with the intervention of acupoint application. As we all know, phenol is often regarded as one of the indicators of pollution in environmental monitoring. It has been demonstrated that environmental pollution can cause follicular atresia, increase follicular depletion, and induce early menopause [28]. And the direct toxic effect of phenol on the reproductive system has also been proved [29]. Herein, phenol was downregulated with the intervention of acupoint application, which proves the efficacy of acupoint treatment.

There is a significant correlation between amino acids and PCOS. The disorder of amino acid metabolism in follicular fluid in PCOS patients may affect the outcome of IVF by changing glucose metabolism and/or inducing inflammation. In addition, normalizing amino acid metabolism in PCOS patients may create a favorable environment for oocyte development [30]. We found that the 2-oxoadipic acid in the biosynthesis of amino acids pathway was downregulated with the intervention of acupoint application. It is worth mentioning that 2-oxoadipic acid belongs to organic acids. The increase in the level of organic acids in the follicular fluid of PCOS patients can cause metabolic disorders and have adverse effects on the pregnancy outcome of IVF-ET [31]. Collectively, the findings suggest that acupoint application may affect the reproductive system by regulating the pathway of amino acid biosynthesis.

Pyrimidine is the structural component of nucleotides, nucleic acids, vitamins, and folic acid. Pyrimidine metabolism plays a critical role in the synthesis of RNA and DNA and formation of proteins [32]. Pyrimidine could cause toxicity to mouse oocytes and early embryos, and affect ovarian function [33], which indicated that acupoint application may have a certain effect on ovarian function and embryonic development by regulating the pathway of pyrimidine metabolism. We found that the pseudouridine in the pathway of pyrimidine metabolism was downregulated with the intervention of acupoint application. As a tumor marker, pseudouridine plays a guiding role in the diagnosis of ovarian cancer and cervical cancer [34, 35]. But we have not yet found any valuable reports about pseudouridine in PCOS.

### 4.1. Limitations

This study had some limitations. First, the dosage of Gn was more cautious in the course of COH in order to avoid the occurrence of ovarian hyperstimulation syndrome (OHSS), which may affect the experimental results to some extent. Second, although some indicators such as IVF pregnancy outcome were improved, the difference was not statistically significant, which may be attributed to the small sample size and short intervention time. In the future, we will appropriately extend the action time of acupoint application and conduct multicenter large-sample randomized controlled trials, with the overarching goal of increasing the accuracy of clinical results.

## 5. Conclusions

A metabolomic study based on ultrahigh performance liquid chromatography-mass spectrometry on the follicular fluid was successfully established and integrally investigated. The metabolites in the follicular fluid were obviously changed in patients with PCOS of the phlegm-dampness type with the intervention of acupoint application. And the related metabolic pathways were identified by KEGG pathway analysis to explain the mechanism of acupoint application. The study confirmed the efficacy of acupoint application on patients with PCOS, which will provide an effective therapy of traditional Chinese medicine for PCOS patients.

## Figures and Tables

**Table 1:** Comparison of clinical background of subjects ((mean ± SEM), ％).

Item	Treatment group (*n* = 30)	Control group (*n* = 30)	Normal group (*n* = 30)	*P*
Age (years)	29.13 ± 3.34	29.57 ± 3.20	30.23 ± 2.76	0.390
BMI (kg/m^2^)	24.85 ± 2.77	25.80 ± 1.71	21.86 ± 3.05^*∗*^^#^	<0.001
Menstrual cycle (days)	49.20 ± 22.84	45.20 ± 18.30	28.77 ± 1.81^*∗*^^#^	<0.001
Years of infertility (years)	3.40 ± 1.98	3.10 ± 1.99	3.40 ± 2.65	0.834
bFSH (mIU/mL)	6.34 ± 1.27	6.57 ± 1.63	6.18 ± 1.34	0.577
bLH (mIU/mL)	8.28 ± 3.92	9.62 ± 3.80	5.73 ± 2.48^*∗*^^#^	<0.001
bLH/FSH	1.27 ± 0.66	1.53 ± 0.67	0.95 ± 0.45^*∗*^^#^	0.002
bE_2_ (pg/ml)	37.27 ± 13.52	37.38 ± 11.61	36.59 ± 10.25	0.962
bP (ng/ml)	0.48 ± 0.30	0.62 ± 0.44	0.72 ± 0.46	0.076
bT (ng/ml)	0.74 ± 0.55	0.68 ± 0.40	0.51 ± 0.28	0.092

*Note.* ^*∗*^ *P* < 0.05 vs. treatment group; ^#^*P* < 0.05 vs. control group.

**Table 2:** Comparison of COH results ((mean ± SEM), ％).

Item	Treatment group (*n* = 30)	Control group (*n* = 30)	Normal group (*n* = 30)	*P*
Gn days (days)	10.10 ± 1.56	11.70 ± 2.28^*∗*^	10.90 ± 1.73	0.006
Total Gn (IU)	2562.92 ± 604.78	2831.25 ± 1101.29	2687.92 ± 849.85	0.497
*E*_2_ levels on hCG day (pg/ml)	3942.83 ± 1224.35	3566.80 ± 1481.01	3866.37 ± 1006.53	0.473
P levels on hCG day (ng/ml)	1.26 ± 0.84	1.14 ± 0.53	1.30 ± 0.58	0.593
LH levels on hCG day (mIU/mL)	1.37 ± 0.91	1.87 ± 1.28	1.24 ± 0.90	0.053
Number of oocytes retrieved	16.93 ± 5.42	15.10 ± 6.40	13.20 ± 4.90^*∗*^	0.041
Number of fertilized oocytes	10.13 ± 3.09	9.27 ± 3.71	7.97 ± 2.72^*∗*^	0.035
Number of available embryos	6.10 ± 2.66	3.13 ± 1.85^*∗*^	3.83 ± 2.68^*∗*^	<0.001
Number of high-quality embryos	1.50 ± 1.46	1.07 ± 1.66	1.27 ± 1.41	0.542
Embryo implantation rate (%)	29.8% (37/124)	27.5% (33/120)	30.3% (36/119)	0.880
Cumulative pregnancy rate (%)	73.3% (22/30)	66.7% (20/30)	70.0% (21/30)	0.853

*Note.* ^*∗*^ *P* < 0.05 vs. treatment group.

**Table 3:** Comparison of phlegm-dampness syndrome integral of the treatment group (mean ± SEM).

Item	Phlegm-dampness syndrome	*T*	*P*
Downregulation day	10.27 ± 4.38	10.597	<0.001
hCG day	6.33 ± 3.30		

**Table 4:** Number of significantly differential metabolites.

Number of significantly differential metabolites Comparison group	72		
	Number of significantly differential metabolites	Upregulated	Downregulated
PCOS-AA vs PCOS-CON	25	19	6
PCOS-NON vs PCOS-CON	34	33	1
PCOS-AA vs PCOS-NON	13	4	9

Note. PCOS-AA: treatment group; PCOS-NON: control group; PCOS-CON: normal group.

**Table 5:** Related information of metabolic pathways.

Pathway name	Map_ID	Corresponding metabolites
Pyruvate metabolism	hsa00620	*DL*-lactate
Nicotinate and nicotinamide metabolism	hsa00760	Nicotinamide
Protein digestion and absorption	hsa04974	Phenol
Biosynthesis of amino acids	hsa01230	2-oxoadipic acid
Pyrimidine metabolism	hsa00240	Pseudouridine

*Note.* Map_ID represented the ID number of metabolic pathway.

## Data Availability

Data and materials are available from the corresponding author upon request.
